# A Double-Blind, Placebo-Controlled Study of Brexpiprazole in the Treatment of Borderline Personality Disorder

**DOI:** 10.1192/bjp.2021.159

**Published:** 2021-11-17

**Authors:** Jon E. Grant, Stephanie Valle, Eve Chesivoir, Dustin Ehsan, Samuel R. Chamberlain

**Affiliations:** 1 University of Chicago, Department of Psychiatry and Behavioral Neuroscience, Chicago, IL USA; 2 Department of Psychiatry, Faculty of Medicine, University of Southampton, USA; and Southern Health NHS Foundation Trust, Southampton, USA

**Keywords:** borderline personality, brexpiprazole, pharmacology, treatment

## Abstract

**Background:**

Borderline personality disorder (BPD) is associated with impaired quality of life and has a number of untoward public health associations. There is no established first-line pharmacological treatment for BPD, and available options are not suitable for all individuals.

**Aims:**

To evaluate Brexpiprazole, which has effects on the dopaminergic and serotonergic systems, for the reduction of BPD symptoms.

**Method:**

Eighty adults with BPD were recruited for a randomized, double-blind, placebo-controlled study. Participants received 12-week treatment with brexpiprazole (1 mg/day for 1 week, then increasing to 2 mg/day) or placebo in a parallel design. The primary efficacy outcome measure was the clinician-rated Zanarini Rating Scale for Borderline Personality Disorder (“ZAN-BPD”). Safety data were collected. Effects of active versus placebo treatment were characterized using linear repeated measures models.

**Results:**

There was a significant interaction between treatment and time on the ZAN-BPD scale (p=0.0031), solely due to differentiation specifically at week 12. Brexpiprazole was generally well tolerated. Secondary measures did not result in statistically significant differences from placebo.

**Discussion:**

Brexpiprazole appears to have some possible effect on BPD symptoms but further studies are needed due to significant effects being evident specifically at the final time point. These findings also need to be viewed cautiously given the small sample size, large drop-out rate, and robust placebo response.

## Introduction

Borderline personality disorder (BPD) is a serious, difficult to treat, psychiatric disorder that causes significant emotional distress, as well as resulting in significant economic burden to health care systems (1–3). A variety of psychotherapies, such as dialectical behavior therapy (DBT), mentalization-based treatment (MBT), and systems training for emotional predictability and problem solving (STEPPS), to name just a few, have shown benefit in reducing many of the core symptoms of BPD (4–6). Healthcare systems, however, often lack the funding and appropriate expertise to implement these treatments, and finding trained therapists has been difficult for many people with BPD (7–8). While research on the use of medication is ongoing, no drug has yet been approved in the United States or elsewhere for the treatment of BPD. Antidepressants, anti-convulsants, and antipsychotics have all been examined (9–17), but current medication options for BPD often provide only partial relief and may have pronounced side effects. Though medications are not currently approved for BPD, many patients are receiving them in clinical practice.

BPD is characterized by a pervasive pattern of affective instability, difficulty with impulse control, and aggressive outbursts. While poorly understood, dysfunctions in the serotoninergic and dopaminergic systems have been implicated in, and considered as possible contributing factors for, these core symptoms of BPD (18–21). Brexpiprazole is a novel serotonin-dopamine activity modulator with partial agonist activity at 5-HT1A and D2/D3 receptors, combined with potent antagonist effects on 5-HT2A, a1B-, and a2C-adrenergic receptors (22–24). In addition, because of low rates of side effects reported in clinical trials for other disorders to date, one would expect brexpiprazole to be fairly well-tolerated in people with BPD. Thus, brexpiprazole may have distinctive properties that make it a promising option to explore in a rigorous clinical trial for people with BPD.

The aim of the present study was to examine the efficacy and safety of brexpiprazole compared to placebo in adults with BPD. We hypothesized that brexpiprazole would reduce the core symptoms of BPD to a greater extent than placebo and would be well tolerated.

## Methods

Eighty individuals aged 18-65 years (mean age=39.7 ± 11.6; females=45 [56.3%]) with a current established diagnosis of BPD (see below for assessment procedures) were recruited from clinic and local advertisements for a 13-week randomized, double-blind, placebo-controlled study in which brexpiprazole or placebo was administered in a 1:1 fashion. All 80 participants had current BPD per Diagnostic and Statistical Manual Version 5 (DSM-5) criteria.

Inclusion criteria for the study were the following: aged 18-65 years; primary diagnosis of BPD; a ZAN-BPD scale total score of at least 9 at study entry; and the ability to understand and sign the consent form. The following were exclusionary: unstable medical illness; schizophrenia or bipolar I disorder; an active substance use disorder; current pregnancy or lactation, or inadequate contraception in women of childbearing potential; subjects with a suicide attempt within the 6-month prior to the baseline visit or significant risk of suicide (in the opinion of the investigator, defined as a “yes” to suicidal ideation questions 4 or 5, or answering “yes” to suicidal behavior on the Columbia-Suicide Severity Rating Scale within the past 6-months); illicit substance use based on urine toxicology screening (excluding marijuana); initiation of psychological interventions within 3 months of screening; use of any new psychotropic medication started within the last 3 months prior to study initiation; previous treatment with brexpiprazole; and cognitive impairment that might interfere with the capacity to understand and self-administer medication or provide written informed consent.

Participants were recruited in the study from the 1st of June 2018 until the 16^th^ of December 2020. The authors assert that all procedures contributing to this work complied with the ethical standards of the relevant national and institutional committees on human experimentation and with the Helsinki Declaration of 1975, as revised in 2008. All procedures involving human subjects/patients were approved by the University of Chicago Institutional Review Board. After a comprehensive explanation of study procedures and an opportunity to ask any questions, all participants provided written informed consent. Participants were compensated 200 USD for time and travel associated with the ten study visits.

### Study Design

Eligible participants were assigned to 13 weeks of double-blind brexpiprazole or placebo treatment (12 weeks of treatment with a 13^th^ week tapering/safety phase). The University of Chicago’s investigational pharmacy – which was independent of the research team – randomized all participants (block sizes of 8, using computer-generated randomization with no clinical information) to either the brexpiprazole or matching placebo in a 1:1 fashion. The study blind was maintained by having placebo and active treatments of identical size, weight, shape, and color, as confirmed by the independent pharmacy.

All participants were assessed each week for the first 2 weeks and then every 2 weeks after that. At week 12, participants were started on a 1-week taper off the medication/placebo. The initial dose of brexpiprazole was 1 mg/d and was increased to 2mg/d by week 2 and then remained at 2mg/d for the remaining 10 weeks of the study. Dosage changes and reductions were not permitted, and participants were discontinued if they experienced intolerable side effects. The dose range was based on safety and efficacy data from previous studies using brexpiprazole. We selected the maximum dose of 2mg/d, which is lower than the FDA approved maximum dose of 3mg/d for major depressive disorder, because of increased potential for side effects at the 3mg/d dose.

All efficacy and safety assessments were performed at each visit. Participants who were not compliant with their use of study medication (defined *a priori* as failing to take placebo or active medication for three or more consecutive days) were discontinued from the study. Due to the COVID19 pandemic, study participants (subjects 62 through 80) were allowed to perform their baseline and follow-up visits online using encrypted videoconferencing with the clinician, instead of in person visits. Blood samples, however, were at the discretion of the study investigator, and where considered medically necessary, the participant had them drawn locally and submitted to the study team.

### Assessments

Those individuals who appeared appropriate for the study, based on telephone screening, were invited for a baseline assessment. The duration of the baseline assessment was approximately 90 minutes and included the following: informed consent, demographic data, concomitant medications, family history data, medical evaluation, urine pregnancy test, urine drug screen, and a psychiatric evaluation (Mini International Neuropsychiatric Interview; MINI) (25).

### Efficacy evaluation

The *a priori* (i.e. determined in the Protocol Document prior to study commencement) primary outcome measure was the change from baseline to week 12 using the total score on the Zanarini Rating Scale for Borderline Personality Disorder (“ZAN-BPD”) (26) (the data from week 13 taper phase was not considered for the efficacy analysis but was used for safety assessments). This semi-structured interview has anchored ratings (0=no symptoms, 4=severe symptoms) on nine items that correspond to the DSM-5 BPD criteria.

Secondary efficacy measures included the self-report version of the ZAN-BPD scale (27), the patient-rated version of the Sheehan Disability Scale (SDS) (28); 24-item Hamilton Depression Rating Scale (HAM-D) (29); and the Hamilton Anxiety Rating Scale (HAM-A) (30).

### Study Withdrawal and Safety

If a participant withdrew from the study, all instruments administered at the baseline visit were completed at the final visit. Safety and tolerability were assessed using spontaneously reported adverse events data, the Columbia Suicide Severity Rating Scale (C-SSRS), and by evaluating premature termination. Safety assessments (sitting blood pressure, heart rate, adverse effects, suicidality, and concomitant medications) were documented at each visit for those who enrolled prior to COVID19 restrictions. Participants who were ever an imminent suicide risk were removed from the study and appropriate clinical intervention (e.g. hospitalization) was arranged. Assessment of side effects was done at each visit.

### Data Analysis

Efficacy analysis involved all visits during the 12-week double-blind treatment phase (up until week 12). All enrolled participants were included in the analyses of baseline demographics and safety according to an intent-to-treat (ITT) principle. For statistical analysis, the full-analysis set was defined as all participants who took at least 1 dose of the study drug and had at least 1 post-baseline primary efficacy assessment. The safety-analysis set was defined as all randomized participants who took at least 1 dose of the study drug and completed at least 1 follow-up safety assessment.

To assess efficacy, we used a linear mixed-effects regression model with the ZAN-BPD total score as the dependent variable. Independent variables included terms for treatment group, study visit, and treatment-by-visit interaction. Imputation was not undertaken for missing data. Correlation between visits for the same participant was modeled using an unstructured correlation or auto-regressive correlation depending on best fit. Residuals and model fit were examined. The pre-specified effect of interest was the treatment-by-visit interaction, specifically the baseline to visit 8 change (i.e. week 12) between groups. SAS v.9.4 (SAS Institute Inc., Cary NC) was used for analysis. All testing was two-sided and p-values <0.05 were considered statistically significant.

The planned study sample size was calculated for the primary endpoint of change from baseline. In power calculation, assuming a similar magnitude of effect seen in other studies of medications for BPD, it was determined that 35 participants were needed in each treatment group to detect a difference with an overall 5% type-I error risk. Given the particularly low rates of adverse events reported with brexpiprazole, as well as its more positive side effect profile in terms of sexual side effects, we expected few drop-outs from the study and therefore a smaller sample was needed (31).

## Results

**Figure 1:**
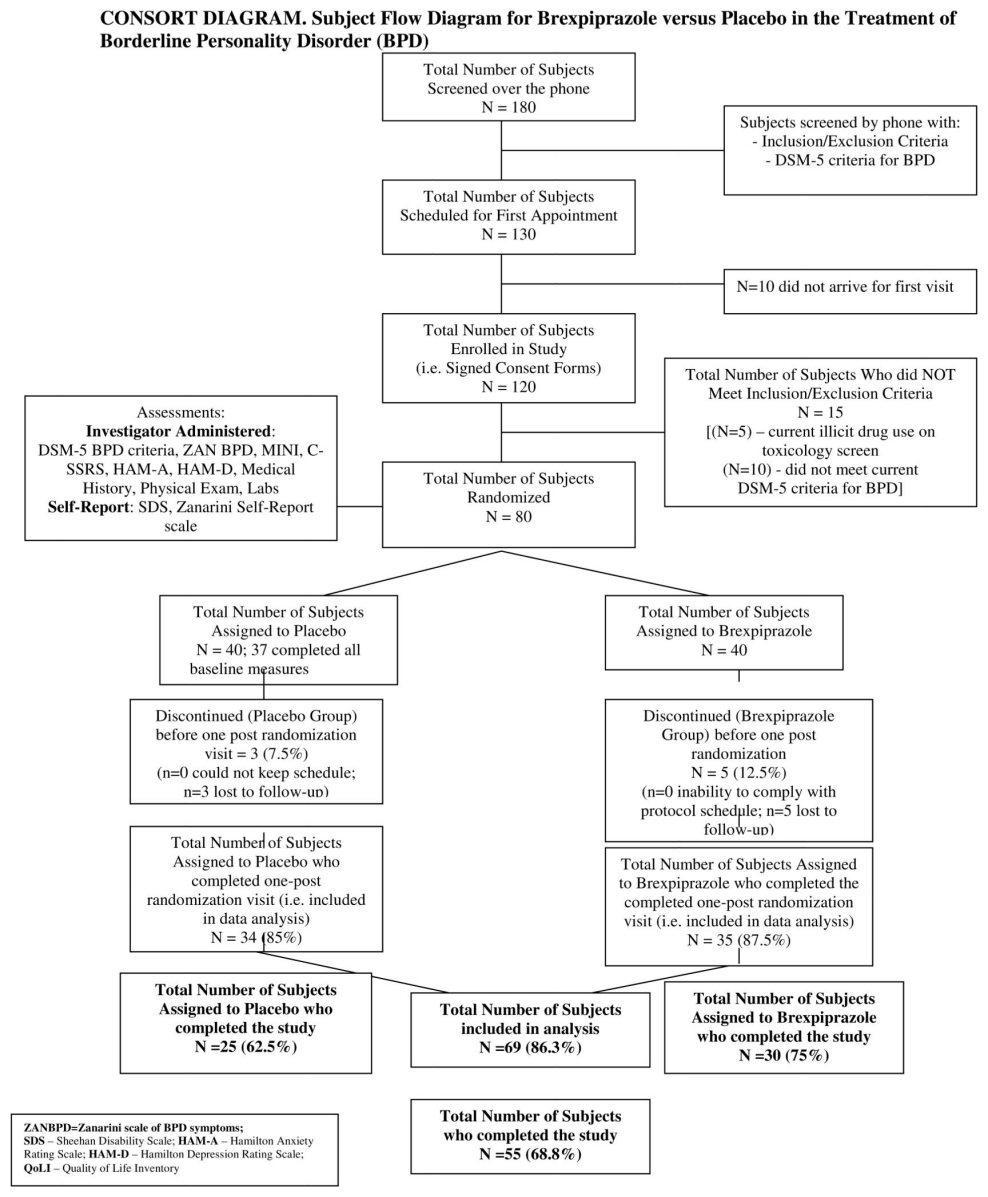


A total of 80 participants signed informed consents, were enrolled, and randomized to brexpiprazole or placebo. Participant flow through the study is presented in Figure 1 (CONSORT Diagram). Of the 80 participants, 40 were assigned to placebo but only 37 completed all baseline measures. Of the 40 assigned to brexpiprazole, all 40 completed the ZAN-BPD scale but only 37 completed the rest of the baseline measures.

Demographic characteristics of subjects at baseline are presented (Table 1). There were no statistically significant differences on demographic variables or clinical measures between groups at study entry. Baseline BPD scores were reflective of moderate severity (15.0 ± 4.5 for placebo group and 14.9 ± 4.4 for the brepiprazole group; Mann-Whitney-Wilcoxon test was used to assess for differences between groups at baseline; p=0.9878). Of the 80 randomized participants 69 (86.3%) returned for at least one post-baseline visit.

Of those assigned to brexpiprazole, 12 (of 40) (30%) were on at least one concomitant psychiatric medication (8 taking an antidepressant, 5 on an anti-epileptic, and 3 on a stimulant) whereas of those assigned to placebo (14 of 40; 35%) were on at least one concomitant psychotropic medication (10 taking an antidepressant, 7 on an anti-epileptic, and 3 on a stimulant).

Of the adults assigned to brexpiprazole, 25 (62.5%) had at least one current comorbid psychiatric disorder (19 with an anxiety disorder, 15 with a mood disorder, 4 with ADHD, and 4 with an eating disorder). Among the people assigned to placebo, 26 (65.0%) had at least one current comorbid psychiatric disorder (19 with an anxiety disorder, 13 with a mood disorder, 1 with ADHD, and 3 with an eating disorder).

A total of 30 of 40 participants (75%) assigned to treatment with brexpiprazole and 25 of 40 individuals (62.5%) assigned to treatment with placebo completed the 12-week trial (Figure 1). Of the 25 participants who failed to complete the study, participants generally withdrew due to perceived lack of efficacy or inability to adhere to the study schedule.

**Figure 2:**
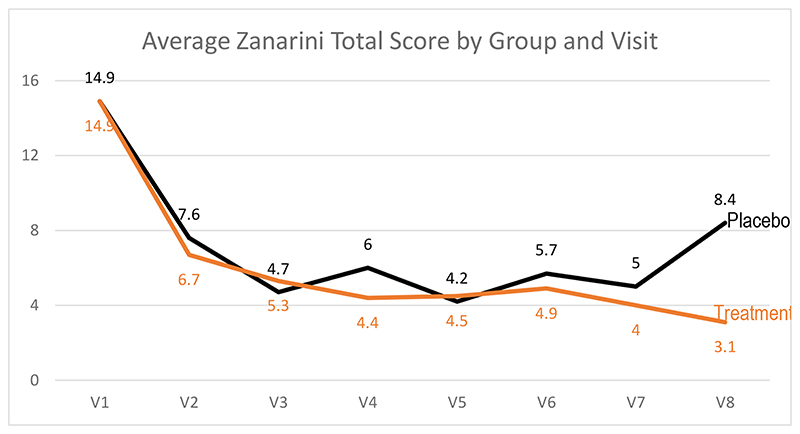
Zanarini Total Score by Visit by Group

The primary outcome variable was ZAN-BPD Total Scores. Figure 2 shows means at each visit by group. The treatment group went from a mean score of 14.9 (4.4) at study entry to 3.1 (3.9) at end of 12 weeks, compared to a change from 14.9 (4.5) for placebo to 8.4 (5.5) at the end of the 12 weeks.

## Regression Model for Zanarini Total Score

There were 69 participants included in this analysis (that had baseline and at least 1 follow-up visit with ZAN-BPD total scores). The model with visits 1-8 included 494 total data points. The statistical model indicated a significant interaction between group and visit number, as well as significant main effects of visit number and of treatment group (Table 2).

**Figure 3:**
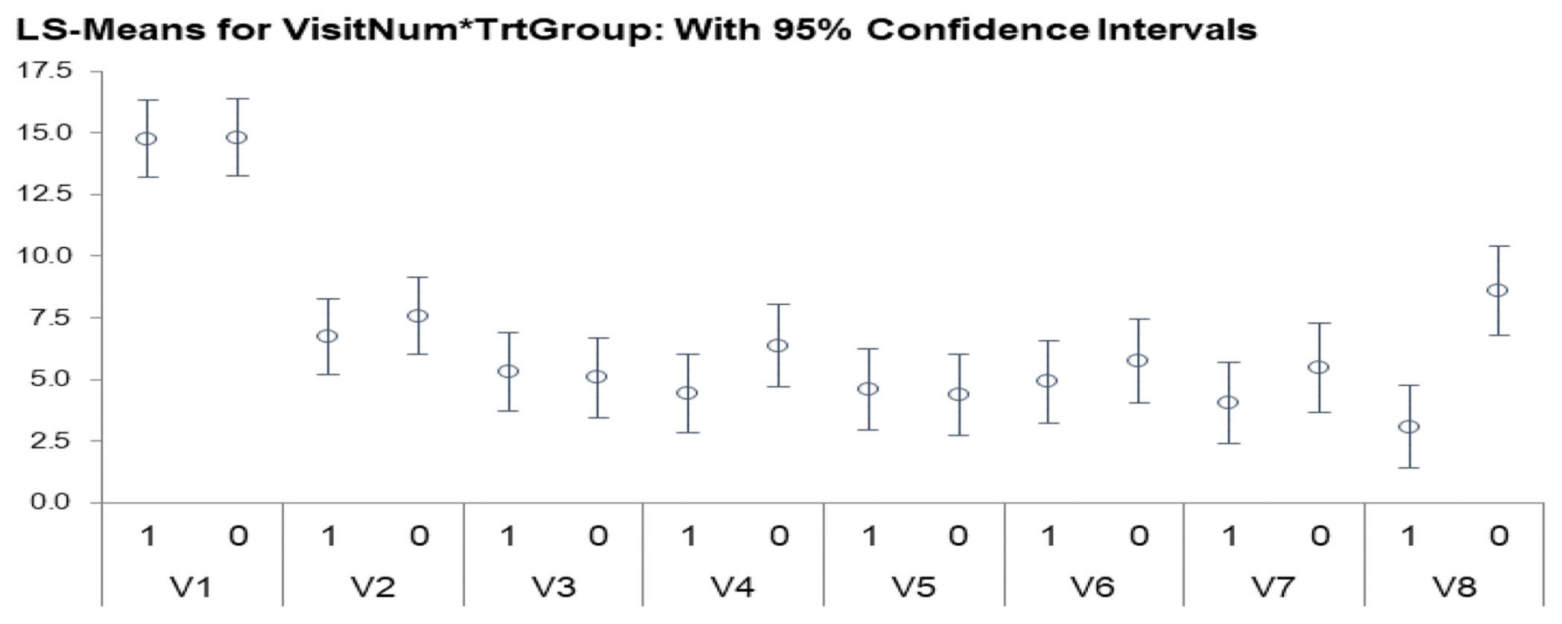
LS-Means for Visit by treatment group. Graph shows, for each time point, LS-Means for the primary outcome measure in the brexpiprazole group (“1”) and placebo group (“0”) respectively. It can be seen that the 95% confidence intervals overlapped at each time point for the groups, except for the final treatment visit (V8) where it can be seen that the placebo-treated subjects had higher Zanari total scores than the actively treated group.

In the model, the treatment group by time interaction term is statistically significant and is driven by the jump in scores at visit 8 in the placebo group. Figure 3 shows that at the various visits, the treatment group and placebo group have similar scores with similar 95% confidence intervals, except for visit 8 in the placebo group. This non-overlapping confidence interval with the visit 8 treatment group CI is driving the significance of this term in the model.

In terms of the secondary measures, there were no significant differences during treatment in terms of anxiety or depression scores.

Adverse event data from the trial showed that brexpiprazole was generally well tolerated with minimal side effects that were of mild intensity. Of the 37 participants assigned to placebo, 19 (51.4%) reported at least one side effect with the most common being nausea (6 participants), fatigue (4 participants), restlessness (3 participants), headaches, hallucinations, and sleep problems (2 participants each), and tremor, sweating and increased appetite (1 participant each). Of those assigned to brexpiprazole, 11 of the 40 (27.5%) reported at least one side effect with the most common being restlessness and dry mouth (3 participants each), nausea and fatigue (2 participants each), and head ache and increased appetite (1 participant each). The groups differed in the number of people experiencing adverse events, due to significantly lower likelihood of adverse events occurring with active treatment compared to placebo (chi-square 4.5979, p=0.0320).

## Discussion

This study showed that brexpiprazole may have had some effect on BPD symptoms but this was primarily due to differentiation from placebo specifically at 12 weeks and therefore further studies are warranted. Having said that, these data make it difficult to imagine that a drug mechanism gave rise to the differences in ZAN-BPD scores at the final visit. Thus, these findings need to be interpreted with some caution. Although the primary outcome measure separated from placebo at the final visit, it had not done so for the first 10 weeks of treatment. This may be due to a robust placebo response in BPD, as evidenced from previous pharmacological trials (11,32,33). This late separation from placebo suggests that perhaps only longer duration trials can provide clear evidence of a strong drug effect, though it is unclear pharmacologically why benefits would not have accrued gradually over time rather than being evident mainly at one time point. Another possibility is that due to the trial nearing its end, medication had some effect on rejection sensitivity versus placebo; thus ZAN-BPD scores tended to increase in the placebo group but less so the active treatment group. Also, given that subjects were informed at visit 8 that their medication would be tapered for the final week, it also remains unclear as to whether some may have exaggerated treatment response (although why in one arm and not the other remains unclear). Again, longer studies may better delineate these findings and confirm a pharmacological response.

The placebo response in this study was quick and fairly sustained for several weeks. This type of robust placebo response is consistent with previous pharmacological studies in BPD (32). Having said that, this type of placebo response can lower statistical power and interfere with interpretation of results. Did the every two week visits provide some sort of nonspecific psychological support to those assigned placebo? One potential solution is to conduct longer trials with less frequent visits. It is unclear whether participants would participate in these types of trials. A placebo lead-in may also be needed to reduce some of this noise and allow for better examination of drug effect.

There are several limitations associated with this study. First, there were some missing data, largely due to switching to an online platform given restrictions from COVID19. Second, the relatively small sample size due to drop out in the early weeks of the study may further call into question whether some of the secondary measures may have been significant if adequately powered. Finally, although well tolerated, the activating side effect of brexpiprazole may have jeopardized the blind potentially, though this seems unlikely given that subjects were more likely to report side effects with placebo.

Despite the limitations, brexpiprazole appears to have had some possible effect on BPD symptoms but further studies are needed due to significant effects being evident specifically at the final time point. Given the strong placebo response future studies should be well powered and long enough to better determine what is a placebo response compared to a true drug effect.

## Figures and Tables

**Table 1:** Characteristics of the BPD Participants at Study Entry

	Placebo Group (n=37)	Treatment Group (n=40)	P-value*
Age, *mean ± SD (median; range)*	28.9 ± 9.6 (25; 19-54)	30.9 ± 12.6 (27; 19-61)	0.6700 (M)
Sex, *n(%) (*N=35 for treatment group)			0.5426 (F)
Female	21 (56.8%)	24 (68.6%)	
Male	12 (32.4%)	9 (25.7%)	
Other	4 (10.8%)	2 (5.7%)	
Race, *n (%)*			0.9736 (F)
American Indian or Alaska Native	0 (0%)	0 (0%)	
Asian	2 (5.4%)	1 (2.7%)	
Native Hawaiian or Pacific Islander	0 (0%)	1 (2.7%)	
Black	6 (16.2%)	7 (2.7%)	
White	22 (59.5%)	20 (54.1%)	
Multiple races	1 (2.7%)	2 (5.4%)	
Unknown	6 (16.2%)	6 (16.2%)	
Ethnicity, *n (%)*			0.3469 ©
Hispanic or Latinx	8 (21.6%)	7 (18.9%)	
Not Hispanic or latinx	13 (35.1%)	19 (51.4%)	
Unknown	16 (43.2%)	11 (29.7%)	
Education, *n (%)* (N=36 for placebo group)			0.3857 (F)
Less than HS diploma/GED	0 (0%)	0 (0%)	
HS diploma/GED	5 (13.9%)	7 (18.9%)	
Some college/Associates	20 (55.6%)	18 (48.7%)	
Bachelors	11 (30.6%)	9 (24.3%)	
Masters+	0 (0%)	3 (8.1%)	
Employment Status, *n (%)*(N=35 for the treatment group)			0.9202 (F)
Full-time	11 (29.7%)	12 (34.3%)	
Part-time	2 (5.4%)	3 (8.6%)	
Student	8 (21.6%)	8 (22.9%)	
Unemployed	15 (40.5%)	11 (31.4%)	
Retired	0 (0%)	0 (0%)	
Other	1 (2.7%)	1 (2.9%)	
Marital Status, *n (%)*			0.7734 (F)
Single	31 (83.8%)	27 (73.0%)	
Married	2 (5.4%)	3 (8.1%)	
Divorced/separated	3 (8.1%)	6 (16.2%)	
Living together/engaged	1 (2.7%)	1 (2.7%)	
Widowed	0 (0%)	0 (0%)	
Zanarini Total Score, *mean ± SD (median; range)*	15.0 ± 4.5 (14; 9-26)	14.9 ± 4.4 (15; 9-26)	0.9878 (M)

* (M) indicates p-value was generated from Mann-Whitney-Wilcoxon test; (C) indicates p-value was generated from chi-square test; (F) indicates p-value was generated from Fisher’s Exact test.

**Table 2:** Regression model examining primary efficacy measure.

Number of participants / visits included	69 / 494			
**Model status**	Converged			
**Model fit: AIC; BIC**	2780.13; 2784.59			
**Effect**	**Num DF**	**Den DF**	**F Value**	**Pr > F**
**Visit Number**	7	411	39.74	<.0001
**Treatment Group**	1	67	4.00	0.0497
Visit Number* Treatment Group Interaction	7	411	3.13	0.0031

## Data Availability

The data that support the findings of this study are available from the corresponding author, [JEG], upon reasonable request. The data are not publicly available due to confidentiality issues.
